# Temporarily trapped: stormwater pond sediment is a key transient sink for microplastic debris

**DOI:** 10.1098/rsta.2023.0029

**Published:** 2025-10-23

**Authors:** Patricia L. Corcoran, Alexa Holland, Kelly Evans, Nina Kozikowski, Rebecca Sarazen

**Affiliations:** ^1^ Department of Earth Sciences, University of Western Ontario, London, Ontario N6G 0J3, Canada; ^2^ Surface Science Western, University of Western Ontario, London, Ontario N6G 0J3, Canada

**Keywords:** microplastics, FTIR, land use, Canada, stormwater, rubber, Great Lakes watershed

## Abstract

Average microplastic (MP) abundances in each of 15 stormwater ponds in London, Canada ranged from 0.7 ± 0.7 particles per gram of dry weight sediment (g^−1^ dw) to greater than 2323 ± 4643 g^−1^ dw. Omitting one wetland cell and one hybrid pond produced a significantly higher median MP abundance for forebay versus main basin samples (*p* = 0.0058), and inlets produced greater mean abundances than outlets and open areas (20 ± 49, 10 ± 17, 4 ± 4 g^−1^ dw, respectively). Considering all 15 ponds, the industrial/commercial and construction ponds produced higher means (684 ± 2387 g^−1^ dw; 19 ± 22 g^−1^ dw) than residential and open counterparts (6 ± 10 g^−1^ dw; 6 ± 11 g^−1^ dw). Fragments were the most common particle type in all but one pond, and fibre concentrations relative to other particle types were greatest in residential ponds. Copious styrene butadiene rubber (SBR) particles in an industrial pond are considered tyre wear due to proximity to manufacturers’ parking areas and loading docks, and an automotive technology training school. Paint/coating chips accounted for 25% of the fragments analysed. With greater MP abundances than in sediment from tributaries, lakes and beaches of the same watershed, stormwater ponds prove to be exceptional transient sinks for MP debris.

This article is part of the Theo Murphy meeting issue ‘Sedimentology of plastics: state of the art and future directions’.

## 1. Introduction

Mismanaged plastic waste has become a common component of Earth’s ecosystem since the mass production of plastic goods for daily use began in the 1950s. Throughout the past 75 years, plastic production increased from 2 million tonnes to over 450 million tonnes [1]. Without modifications to the life cycle of plastics, it is estimated that in aquatic systems alone, the amount of plastic waste could triple by 2040 [2]. The continued accumulation of plastic debris in the environment is, however, not always visible because much of it is in the form of microplastics (MPs). MPs, which are particles of plastic less than 5 mm in diameter, are considered primary when they are intentionally produced to be less than 5 mm, and secondary when they are eroded following degradation of larger plastic products. The subsequent increase of MPs in the environment relative to macroplastic items (greater than 5 mm) results in the former’s greater availability for ingestion by a variety of organisms [3]. MPs have been studied in numerous environmental matrices, such as surface water [4], air [5], snow [6], ice [7], soil [8], sediment [9] and biota [10]. We have found, however, that in contrast to common geographic compartments, such as oceans and lakes, at the time of writing, fewer than 20 journal articles focus on MPs in stormwater pond sediment.

Stormwater ponds are designed as small and shallow basins, generally within urbanized areas, that are used to capture surface runoff during rain events. The runoff often contains physical and chemical pollutants that are allowed to settle to the pond bottom before the water escapes or is intentionally released into natural waterbodies [11,12]. The ponds also serve to prevent flash flooding of urbanized areas and natural waterbodies and to retain nutrients through biological processes [13]. As catchment structures, stormwater ponds also accumulate sediment and require dredging when the basin becomes too full according to the standards set in different jurisdictions. Stormwater runoff is an important pathway for the deposition of MPs from land-based sources to aquatic environments, but it is greatly influenced by the factors controlling plastic pollution on land [14]. Some of these factors include proximity to high traffic areas that leads to an increased number of road wear particles, building construction activities that create paint and insulation microparticles, and high population density resulting in abundant fibres and glitter particles [15–17].

Previous studies of MPs in the sediment of stormwater ponds showed maximum MP concentrations as high as 127 986, 154 709 and 950 000 particles per kilogram of dry weight sediment (kg^−1^ dw) [13,18,19, respectively]. These abundances are not entirely comparable because MPs of different sizes were examined. Nonetheless, the results to date suggest that stormwater pond sediment is an important sink for MP debris.

We sampled, processed and examined 86 sediment samples from 15 stormwater ponds within the city of London, Ontario, Canada. Our objectives were to (i) compare the abundances and main types of MPs between ponds, (ii) investigate the relationships between MP abundances and within-pond location, pond orientation, last dredged date and local land use, and (iii) compare the abundances of MPs in 53 to 2000 µm size sediment of stormwater ponds, lakes and rivers of one regional watershed.

## 2. Material and methods

### (a) Study location

**Figure 1.**
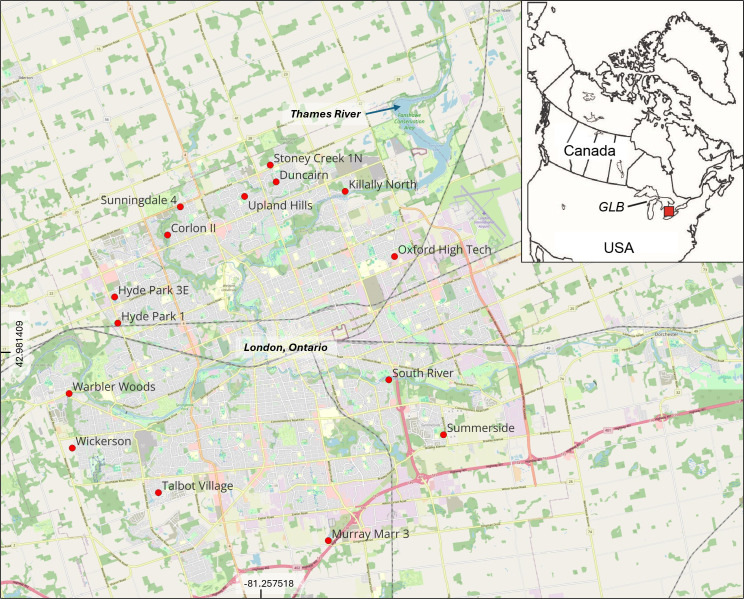
Location of the 15 studied stormwater ponds in London, Canada. Inset map displays the location of the Thames River watershed (shaded square) within the Great Lakes Basin (GLB).

**Figure 2.**
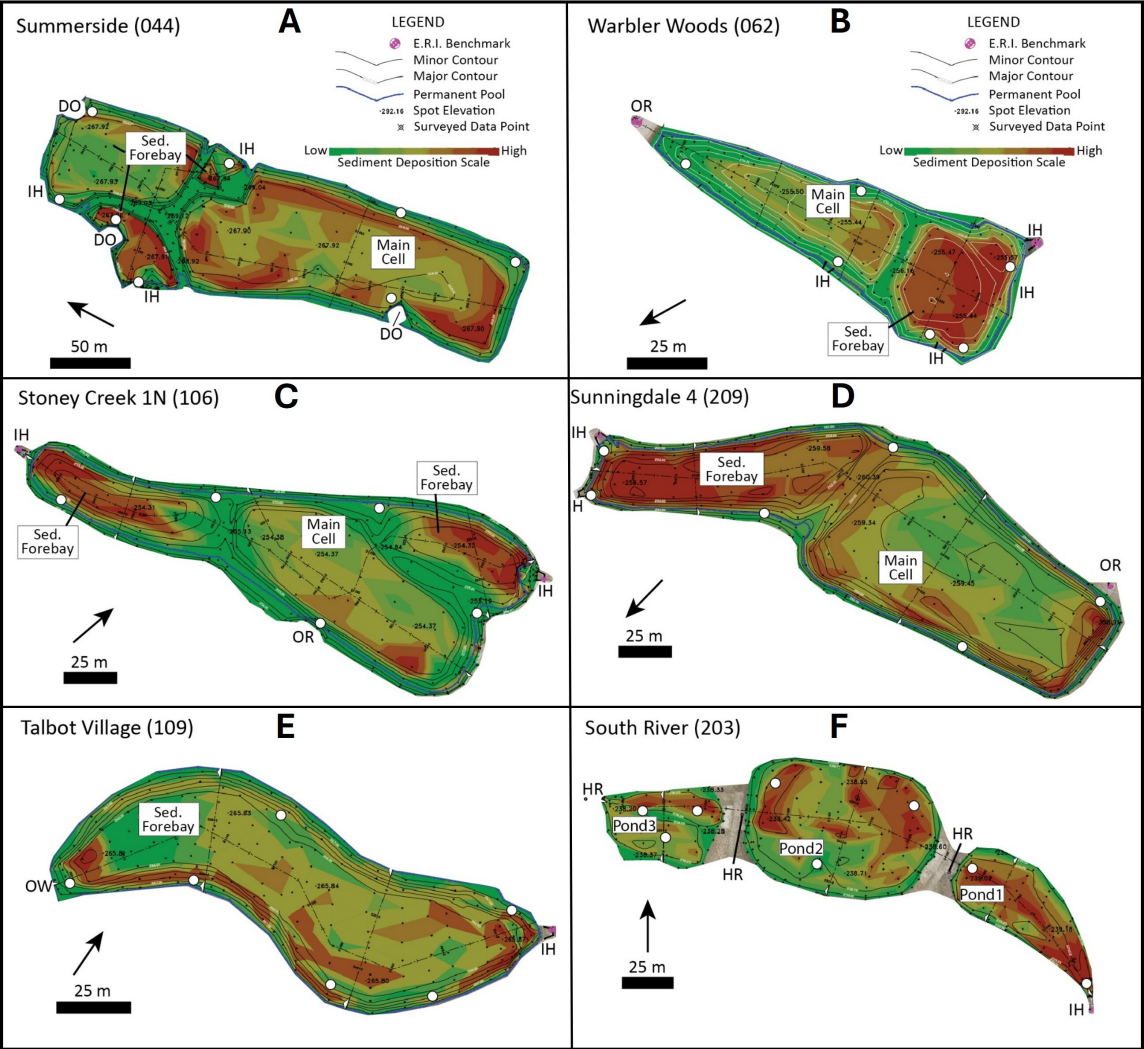
Examples of the stormwater ponds sampled in London, Canada. Wet retention ponds (A) Summerside, (B) Warbler Woods, (C) Stoney Creek 1N and (D) Sunningdale 4 contain forebays and main cells. Talbot Village retention pond (E) contains only a forebay, and South River detention pond (F) contains Hickenbottom risers between each pool. White circles indicate sample locations and black arrows point to north. IH, inlet headwall; DO, drain outlet; OR, outlet riser; OW, outlet weir. Modified from the 2019 engineering specification diagrams drafted by Ecosystem Recovery Inc. for London’s stormwater management sediment survey and sediment removal forecasting project.

The city of London is located within the Thames River watershed of the 244 106 km² Laurentian Great Lakes Basin, which straddles the border between Canada and the United States. Bottom sediment samples from 15 stormwater ponds in London were collected during autumn of 2022 (figure 1). At the time of sampling, Murray Mar 3 (076) was last dredged in 2005, Killaly North (093) and Hyde Park 1 (063) in 2012, Sunningdale 4 (209), Wickerson (074), Summerside (044) and South River (203) in 2013, the main basin of Hyde Park 3E (064) in 2014, Oxford High Tech (030) and Upland Hills (047) in 2015, Duncairn (009), Stoney Creek 1N (106), Corlon II (069), Warbler Woods (062) and Talbot Village (109) in 2016, and the forebay of Hyde Park 3E (064) in 2021 (table 1). Twelve of the 15 studied stormwater ponds are wet retention ponds composed of 1−2 forebays, which house one or more inlets, and a main basin, where one or more outlets are located (figure 2A-D). The forebay is typically deeper than the main basin, as it is designed to trap most of the incoming stormwater runoff and sediment [20]. The main basin is relatively shallow and receives sediment either during flood events or once the forebay is filled. Talbot Village is also a wet pond, but it contains no main basin (figure 2E). In contrast, South River is considered a dry or hybrid pond, which holds water temporarily during flood flow (figure 2F). The water flows from the eastern to western pools through a terraced underground drainage system linked by Hickenbottom riser control structures. The result is that, unlike wet ponds, the South River pond is dry many times of the year (electronic supplementary material, fig. S1a). Oxford High Tech also differs from the other ponds in that it contains a wetland cell and a dry cell, with the former promoting growth of aquatic vegetation in shallow water (electronic supplementary material, fig. S1b).

**Table 1.** Summary characteristics of the fifteen stormwater ponds studied in London, Canada. The total and average MP abundances per pond are based on the results of 86 sediment samples. Res.: Residential; Constr.: Construction; Ind./Com.: Industrial/Commerical.

pond	total MPs (g^−1^ dw) per pond	average MPs (g^−1^ dw) per pond	land use	orient.	last dredged	# of houses within 100m	basin
Duncairn	23.9	6.0 ± 9.1	res.	NW-SE	2016	66	main
Oxford High Tech	9290.1	2322.5 ± 4642.8	res.	NE-SW	2015	15	forebay
Summerside	169	21 ± 24	constr.	N-S	2013	36	main
Upland Hills	17.3	3.5 ± 6.1	res.	E-W	2015	60	main
Warbler Woods	15.6	2.6 ± 31	res.	NE-SW	2016	20	forebay
Hyde Park 1	20.1	4.0 ± 4.0	open	E-W	2012	9	forebay
Hyde Park 3E	280	40.1 ± 91.7	ind./com.	N-S	2021	107	forebay
Corlon II	24.7	3.5 ± 4.5	es.	NE-SW	2016	32	main
Wickerson	62.5	10.4 ± 17.2	open	NE-SW	2013	7	forebay
Murray Marr 3	2.7	0.7 ± 0.7	open	NE-SW	2005	0	main
Killaly North	30	6.0 ± 6.8	res.	E-W	2012	54	forebay
Stoney Creek 1N	36.4	7.3 ± 9.8	res.	NE-SW	2016	62	forebay
Talbot Village	40.5	6.8 ± 5.6	res.	E-W	2016	42	main
South River	97.5	12.2 ± 9.3	res.	E-W	2013	13	n/a
Sunningdale 4	37	6.2 ± 4.8	res.	E-W	2013	46	forebay

### (b) Sediment sampling

The number of samples collected from each pond depended on the number of inlets and outlets, pond size and accessibility. For example, Summerside pond is composed of four basins, three inlets and three outlets, and thus, eight samples were collected (figure 2A). Warbler Woods has only two basins and was therefore sampled for sediment in six locations (figure 2B). Sample sites were selected to achieve as regular spacing as possible along the periphery of each pond, but selection was also controlled by bank accessibility, water depth and presence or absence of sediment finer than pebble size.

Bottom sediment was sampled from each site using a stainless steel petite ponar grab sampler, 16 × 14.5 cm in size. One person waded out into the pond and lowered the ponar into undisturbed bottom sediment. Samples were collected from water depths between 50 and 125 cm. The distance from the shoreline varied depending on the slope of the pond banks. Each sediment sample was emptied on to a stainless steel tray and approximately 500−600 ml of sediment was scooped with a stainless steel trowel and placed into a glass jar. Each jar was covered with aluminium foil and a metal lid for transport to the laboratory. All samples were stored in a refrigerator for later processing.

### (c) Sample processing

Sample processing followed a similar procedure to that used by Ballent *et al*. [21] and Corcoran *et al*. [22]. Each sediment sample was emptied into a 53 μm stainless steel sieve for wet sieving using reverse osmosis (RO) water. This process removed the clay and less than 0.053 mm size silt particles. Removal of the fine particle fractions reduces clay flocculation while drying in the oven, which would cause difficulties with density separation. All samples were dried at 60°C for at least 48 h. For samples with minor clumping, the sediment was crushed gently using a ceramic mortar and pestle. Using a metal mini sample splitter, each sample was divided into two subsamples. One was stored in a glass jar, and the second underwent further processing. Subsamples containing grains larger than medium sand were dry sieved using a mesh size of 2.0 mm. Grains greater than 2 mm were not used because they clog the stopcock in the separatory funnels. The grain size fraction between 0.053 and 2 mm was weighed, then emptied into a glass beaker containing 250 ml of sodium polytungstate solution with a specific gravity of 1.5 g cm^−3^. Each sample was magnetically stirred for 2 min and poured into a glass separatory funnel. Once the grains had settled completely, the non-buoyant material was filtered out through the stopcock. The remaining supernatant, consisting of MPs and organic matter, was filtered through 25 μm quantitative fast flow filter paper, drained through a 53 μm stainless steel sieve with a diameter of 7.5 cm, and rinsed thoroughly with RO water. The MPs and organics were transferred to a glass Petri dish using RO water, covered with aluminium foil, and placed back into the oven to dry at 60°C before microscopic examination.

### (d) Microplastic identification

The material in each Petri dish was examined using a stereomicroscope at magnifications of 3.75× to 258× to separate suspected MPs from any organic material. Suspected MPs were characterized according to their colour and morphology (fragment, fibre and bead). The fragment category included films, but glitter and black rubber particles were given their own category. Each particle was removed from the Petri dish using stainless steel tweezers or a dental pick and was placed on double-sided tape on a labelled glass slide. A total of 514 MPs were selected for Fourier transform infrared spectroscopy (FTIR) using a random number generator. At Surface Science Western, in London, Canada, the particles were transferred to a diamond compression cell, condensed and analysed using a Bruker Tensor II spectrometer in transmission mode, with a Hyperion 2000 microscope. Particles less than 2 mm × 2 mm were analysed using a micro-attenuated total reflectance attachment equipped with a germanium crystal. Spectra were collected from 4000 to 600 cm^−1^ with a resolution of 4 cm^−1^. The results were baseline corrected and corrected for possible contamination from water vapour, carbon dioxide and the adhesive tape on which the MPs were mounted.

### (e) Quality control and contamination

Consistent efforts were made to minimize sample contamination. All samples were processed and examined in a laboratory with three air filter systems to minimize airborne MP contamination. Sample splitting and density separation were conducted under a fume hood, and dry sieving was done under a metal cover. All laboratory materials were made of stainless steel, aluminium or glass, except for the filter papers, which are composed of white translucent cellulose. Cellulose fibres derived from the papers were easily identified in the samples and were not included in the counts. While stored in aluminium pie plates, beakers and separatory funnels, the samples were covered with aluminium foil. Cotton lab coats were worn during processing and microscopic examination. One field blank was collected from each pond (*n* = 15) by leaving a glass jar open while the sediment sample was exposed to the air. Fourteen processing blanks and nine microscope blanks were collected by leaving uncovered glass Petri dishes next to the sink during wet sieving and next to the microscope during examination. The average number of MPs in 15 field blanks (0.87) was added to the average number of MPs in eight processing blanks (1.0) and the average number of MPs in 15 microscope blanks (0.67). The blank total (three), composed entirely of fibres, was then subtracted from the initial number of fibres identified in each sample (electronic supplementary material, table S1).

### (f) Statistical analysis

All graphs were made using Microsoft Excel for Microsoft 365 MSO. All statistical analyses were conducted using R v. 4.3.1 [23]. The *p*-values were determined by site and by pond, with a significance level of 0.05, using the Kruskal–Wallis rank sum test (abundance versus location; abundance versus land use; abundance versus last dredged) and the Wilcoxon rank sum exact test (abundance versus basin). Figure 1 was created in QGIS v. 3.34.3.

## 3. Results

### (a) Fourier transform infrared spectroscopy and microplastic abundances

**Figure 3.**
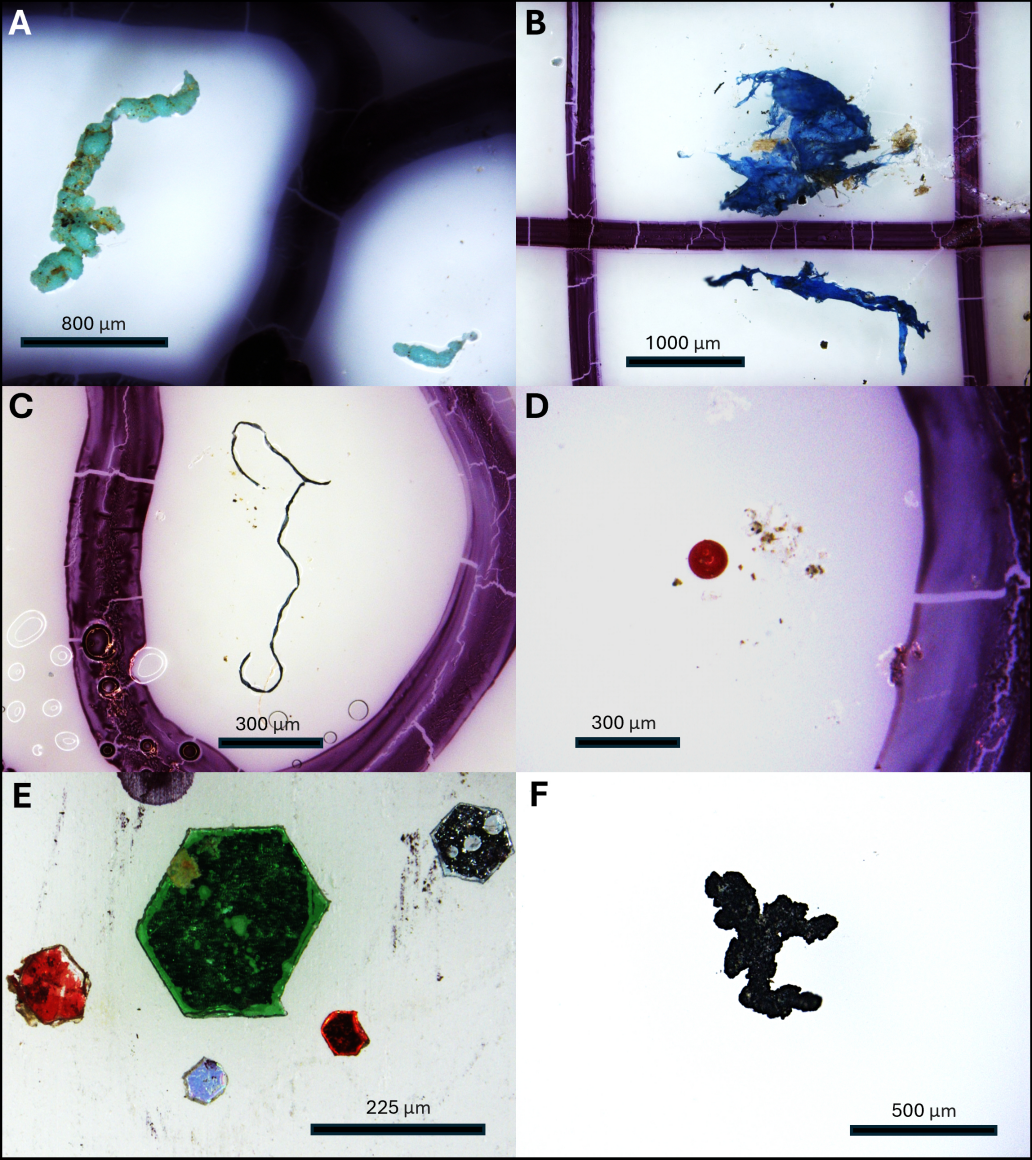
Images of the different MP categories in the present study. (A) Two particles classified as fragments, (B) Film particles grouped into the fragment category, (C) a fibre, (D) a bead, (E) glitter particles and (F) a black rubber particle.

A total of 24 561 potential MPs were initially identified microscopically (electronic supplementary material, table S1; figure 3). Following blank subtraction of fibres, the total became 24 332. FTIR was conducted on 357 fragments (including films), 108 fibres, 19 beads, 10 glitters and 20 rubbers. Of the 357 fragments, 95% were polymers with the most common being polyethylene (PE, 32%), polypropylene (PP, 19%), acrylics (9%), alkyds (7%), polyurethane (PU, 6%), polyvinyl chloride (PVC, 6%) and polyethylene terephthalate (PET, 2%) (electronic supplementary material, table S2). Paint-related polymers (acrylics, alkyds, epoxy, PU) accounted for 25% of the total fragments analysed. Of the 108 fibres analysed, 87 (79%) were polymers with the most common being PET (57%), PP (22%), polyacrylonitrile and acrylonitrile (11%) and nylon (6%) (electronic supplementary material, table S2). Seventy-three per cent of the beads analysed were composed of various polymers, and all 10 glitters (100%) were composed of PET. We analysed 20 black rubber particles using FTIR, but identification was challenging because the carbon black within the particles absorbed infrared light, thereby masking spectral peaks. Eleven rubber particles had weak signals, and thus their composition is unknown. The remaining nine particles were all determined to contain styrene butadiene rubber (SBR; electronic supplementary material, table S2). All black rubber particles in the 86 examined samples had the same textural features—black, with ragged edges and spongy textures (figure 3F), and thus they were all considered to be SBR. The total number of MPs in each category was then corrected by multiplying them by the percentage of analysed particles that were proven to be polymers. For example, the total number of fragments initially identified in sample 009-1 was 115, and this number was multiplied by 0.95 to give a corrected total of 109 fragments. Following FTIR normalization of all particle types, the total number of MPs in all 15 ponds was determined to be 23 321 (electronic supplementary material, table S1).

**Figure 4.**
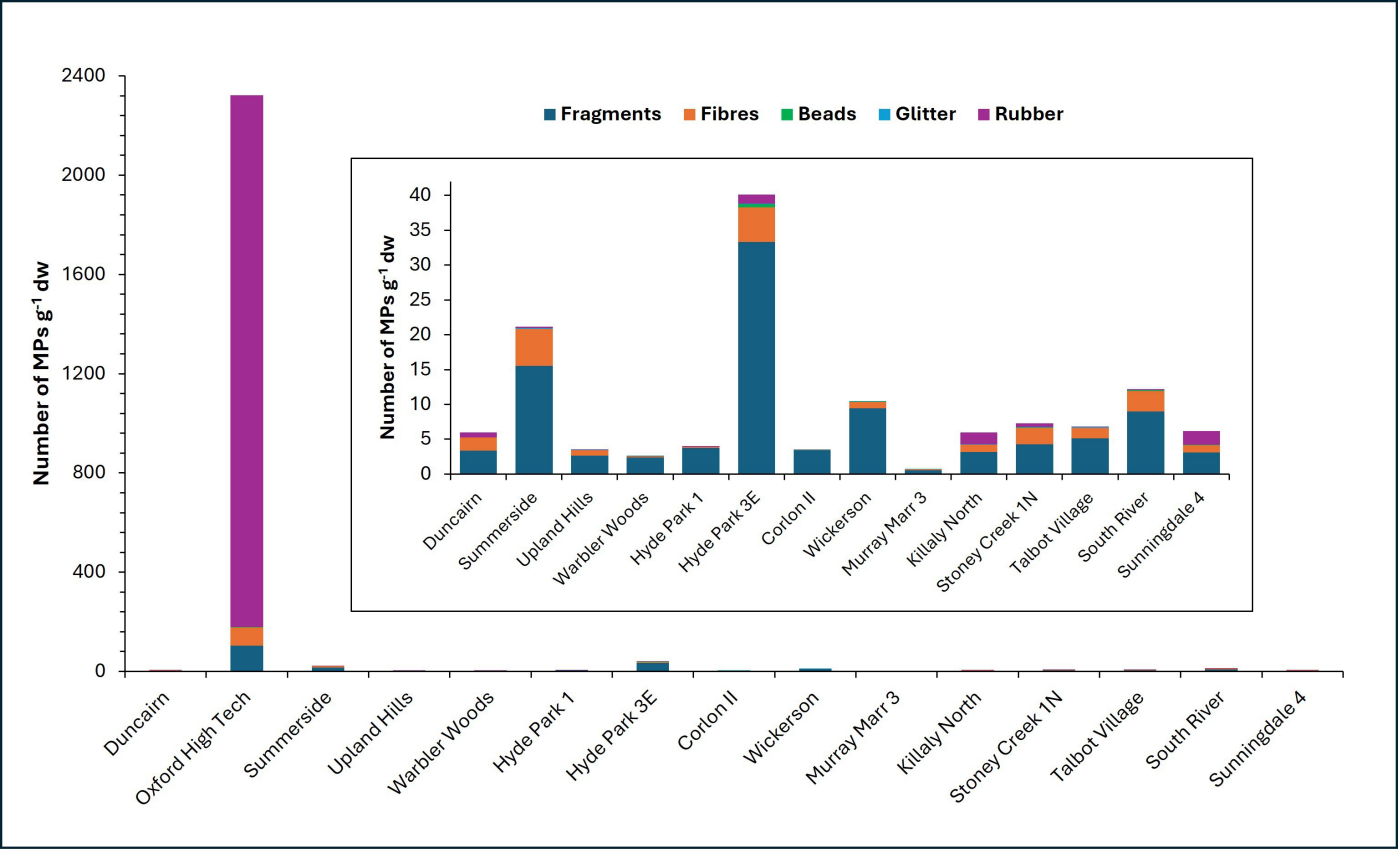
MP types and abundances from 86 sediment samples of 15 stormwater ponds. Inset graph is a version of the main graph without the MP abundances in Oxford High Tech samples.

MP abundance for each sample was calculated based on the corrected particle total and the mass of the sediment sample following wet sieving (electronic supplementary material, table S1). The MP abundances, stated as g^−1^ dw (number of particles per gram of dry weight sediment), range from 0 g^−1^ dw for sample 069-1 of Corlon II to at least 9287 g^−1^ dw for sample 030-4 of Oxford High Tech, based on a minimum SBR count of 12 000 for the latter (figure 4). With omission of sample 030-4, the maximum MP abundance was 247 g^−1^ dw (064-2 of Hyde Park 3E pond). Excluding the anomalous Oxford High Tech pond reveals that the average abundances of MPs in each pond vary from 0.7 ± 0.7 g^−1^ dw (Murray Marr 3) to 40 ± 92 g^−1^ dw (Hyde Park 3E). Given the nature of Oxford High Tech (wetland cell) and South River (hybrid/dry pond), and the anomalously high abundance of SBR particles in Oxford High Tech sample 030-4, the two ponds are not included in the graphs of abundances versus within-pond location, pond orientation and local land use.

### (b) Microplastic abundances and controlling factors

**Figure 5.**
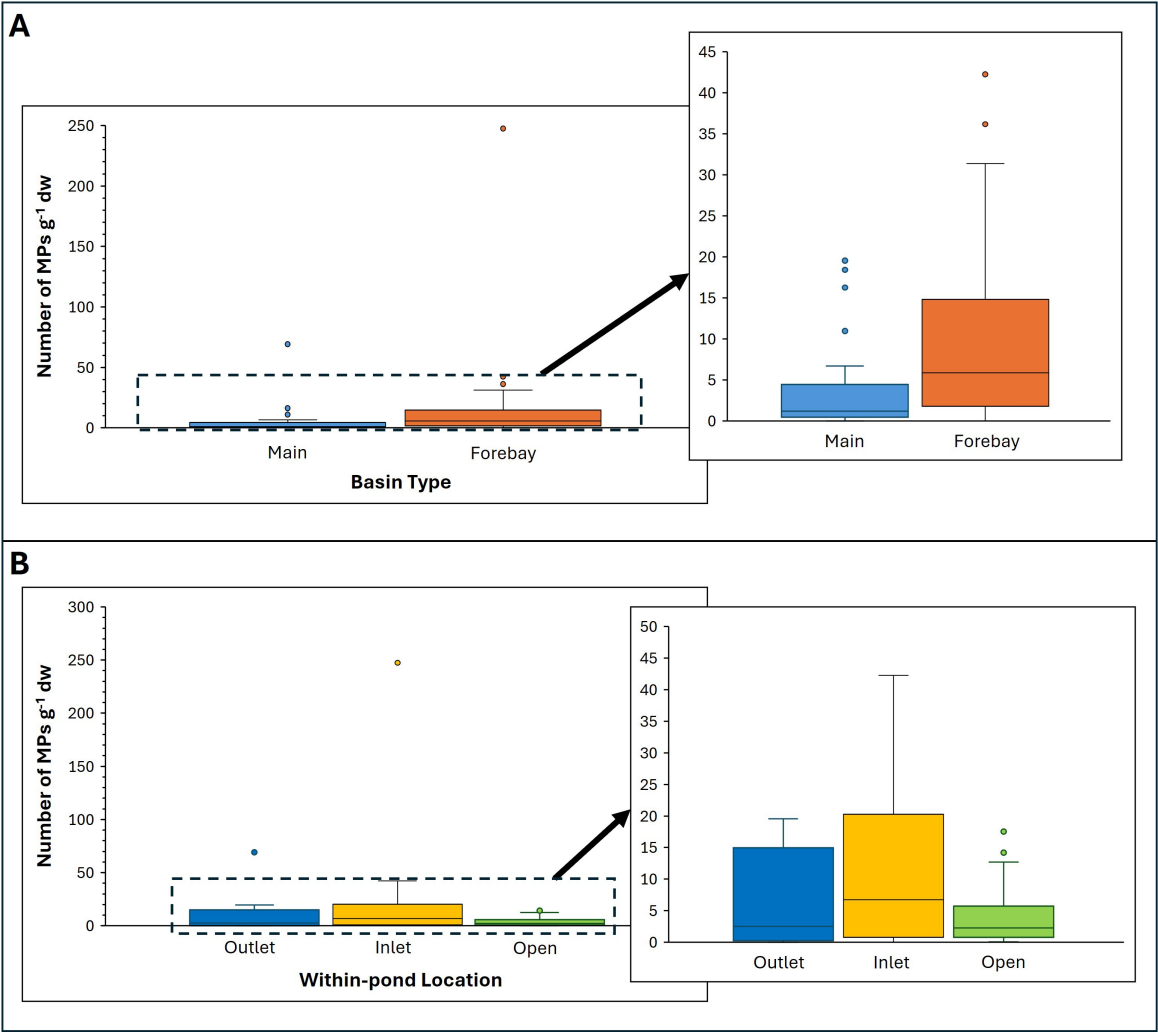
Box and whisker plots of MP abundances in all samples from the (A) main basins and sediment forebays and (B) inlets, outlets and open areas of the ponds. The horizontal line inside each box represents the median.

We attempted to determine if MP abundance is related to within-pond basin type. Figure 5A shows that the median MP abundances are greater for the forebay samples compared to the main basin samples. A Wilcoxon rank-sum test indicates that the difference between the basins is significant (*p* = 0.0058). Comparing abundances in samples from the pond inlets, outlets and open areas shows that the median MP abundances are greatest near the pond inlets (figure 5B), but according to a Kruskal–Wallis test, the differences are not significant (*p* = 0.4453).

**Figure 6.**
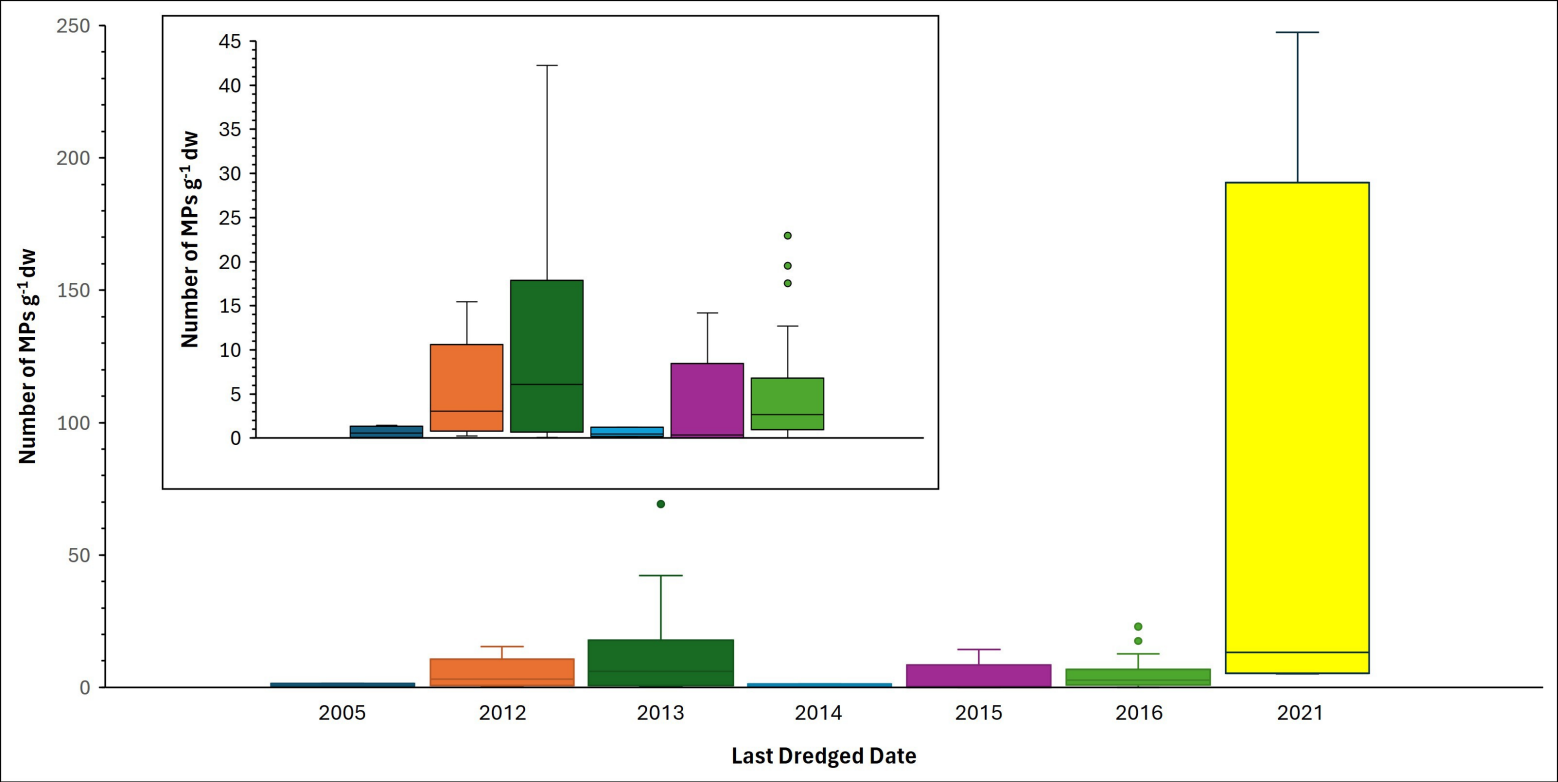
Box and whisker plots of MP abundances in the 13 wet, retention stormwater ponds versus the last dredged date of each pond. Inset plot is a version of the main graph without the ponds last dredged in 2021.

**Figure 7.**
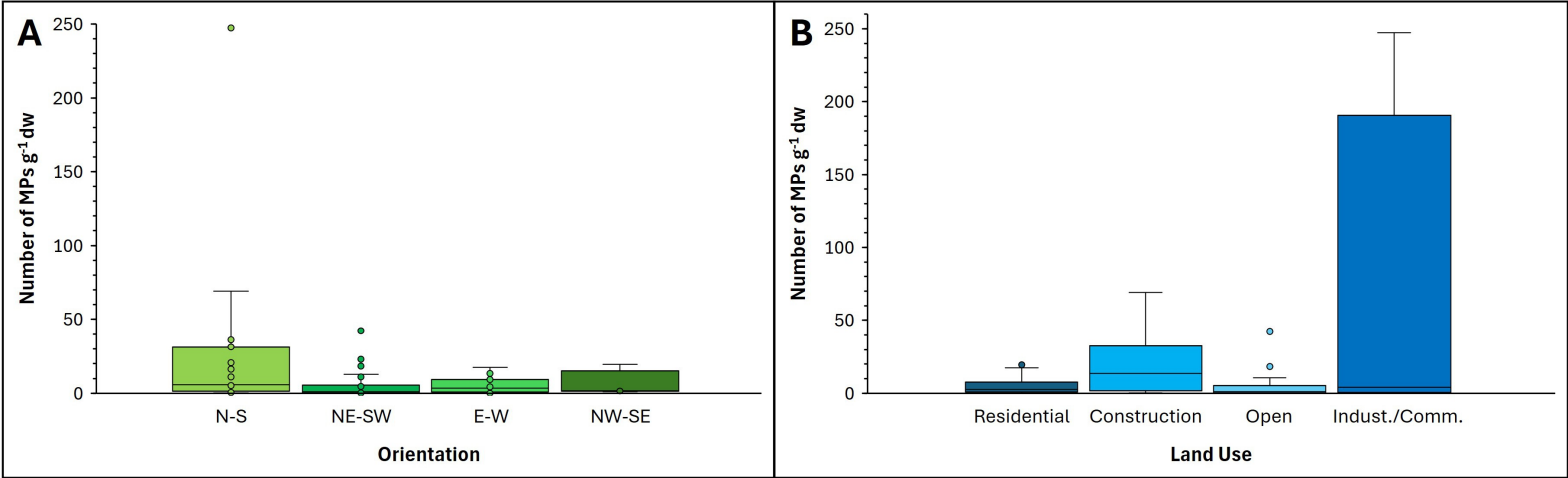
Box and whisker plots of MP abundances in sediment samples from thirteen stormwater ponds versus: (A) pond orientation and (B) land use. Note that the upper outlier value (circle) for the industrial/commercial (indust./comm.) land use category would be 9287 g^−1^ dw if the Oxford High Tech pond samples were included.

No association was determined between last dredged date and MP abundance (figure 6; *p*‐value = 0.1723). We also tested for an association between MP abundance and pond orientation (N-S, NE-SW, E-W, NW-SE). Ponds with their long axes oriented in a N-S direction displayed the highest overall median MP concentration (figure 7A), but the difference between the four directions was not significant according to a Kruskal–Wallis rank sum test (*p*‐value = 0.0737). We also designated each pond according to four local land use types: (i) *residential*: houses within 50−100 m of the pond periphery and vegetation along part of the pond (electronic supplementary material, fig. S1c), (ii) *construction*: houses and building construction within 50−100 m of the pond periphery, (iii) *commercial/industrial*: houses and industrial and/or commercial buildings within 50−100 m of the pond periphery (electronic supplementary material, fig. S1df), and (iv) *open*: 10 or fewer buildings within 50−100 m of the pond periphery (electronic supplementary material, fig. S1e). The ponds surrounded by construction activities and commercial/industrial activities contained the greatest median MP abundances (figure 7B), but the differences between all four land use types were not statistically significant (*p*‐value = 0.09325).

### (c) Microplastic types

**Figure 8.**
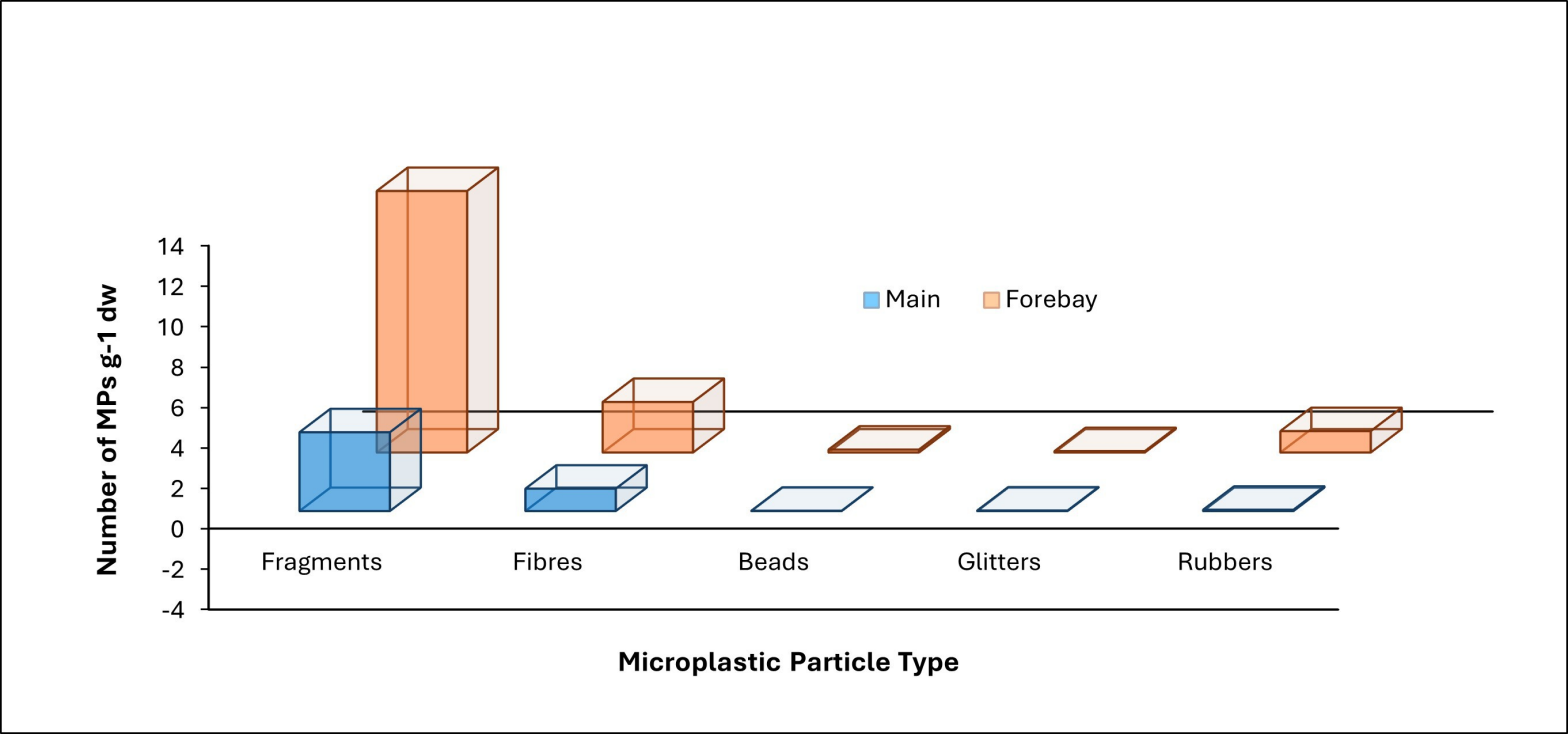
Three-dimensional bar graph displaying the relative abundances of each particle type in the forebay versus main basin samples in the 13 wet, retention ponds.

Within-pond location (basin type) was the only variable for which a *p*‐value less than 0.05 was determined when compared with MP abundances, and therefore, associations between different MP particle morphologies and basin type were investigated. Following removal of the Oxford High Tech and South River samples, the sediment forebay samples contained more than 17× the rubber, 10× the beads, 3× the fragments and glitter and 2× the fibre concentrations than the main basin samples (figure 8). Eighty-five of the 86 sediment samples collected for this study contained a greater proportion of fragments than fibres, beads, glitter and black SBR (figure 4). Oxford High Tech sample 030-4 contains more SBR particles than any other type, with a minimum of 9286 g^−1^ dw (electronic supplementary material, table S1). This sample also contains the greatest overall abundances of fragments (411 g^−1^ dw), fibres (292 g^−1^ dw), beads (8 g^−1^ dw) and glitter (4 g^−1^ dw). Of the remaining 85 sediment samples, the highest abundances of fragments (205 g^−1^ dw), fibres (30 g^−1^ dw) and beads (3 g^−1^ dw) were found in Hyde Park 3E sample 064-2, whereas Summerside sample 044-3 contained the greatest glitter abundance (1 g^−1^ dw) and Sunningdale 4 sample 209-2 contained the highest concentration of SBR particles (12 g^−1^ dw). Of the 15 ponds, only Murray Marr, Wickerson and Corlon II contained no black SBR in their samples (figure 4).

## 4. Discussion

### (a) Comparisons of microplastic abundances in stormwater pond sediment

The total average abundance of MPs in 86 bottom sediment samples from 15 stormwater ponds in London, Canada, is 118 ± 995 g^−1^ dw, with an upper limit of SBR particles set to 12 000. Although we sampled approximately 500−600 ml of sediment at each site, the masses of our samples varied considerably following wet and dry sieving, and we also kept one half of each sample for future grain size analysis. The sample dry weights thus range from 0.18 to 594.73 g (electronic supplementary material, table S1). Although the concentration of MPs in sediment is mainly reported as number of particles per kilogram of dry weight sediment (kg^−1^ dw), we chose to report abundances based on grams rather than kilograms due to some of the very low sample masses. If the average MP abundance is extrapolated to kg^−1^ dw, the resultant value of 118 001 is much greater than the averages determined from MPs in stormwater pond sediment of São Paulo, Brazil (57 542 kg^−1^ dw [24]), Aarhus, Denmark (44 383 kg^−1^ dw [19]) and Memphis, USA (45 800 kg^−1^ dw [25]).

Of the 45 ponds thus far investigated for MPs in sediment, only the present project considered a total of 15 with four or more samples from each pond (electronic supplementary material, table S3). Comparing the average MP abundances in this study with the 30 other ponds published in the literature demonstrates that Oxford High Tech contains one of the highest average concentrations of MPs reported thus far. Olesen *et al*. [13] determined an average abundance of 950,000 kg^−1^ dw from a pond in Viborg, Denmark, but the authors examined sediment grain sizes between 10 and 5000 μm; a much greater size range than the 53−2000 μm that we examined. The London, Canada ponds would therefore contain greater abundances of MP particles if all sediment grain sizes (1−5000 μm) were investigated. For example, Rasmussen *et al*. [26] determined an average abundance of 34 004 900 kg^−1^ dw from the Mariager pond in Denmark, and their studied grain sizes were between 1 and 5000 μm. The cross-study variations in grain sizes, number of ponds and number of samples from each pond demonstrate how direct comparisons between similar MP studies are challenging.

### (b) Stormwater ponds as critical transient sinks for microplastics

The overall average abundance of MPs in the 86 sediment samples is much greater than average abundances reported from 53 to 2000 μm size sediment samples of the Thames River, Canada (304 kg^−1^ dw [22]), Lake Erie nearshore and offshore (213 kg^−1^ dw [27], 531 kg^−1^ dw [28]) and beaches (85 kg^−1^ dw [27]), Lake Ontario nearshore (977 kg^−1^ dw) and tributaries (6272 kg^−1^ dw) [21], Lake Huron offshore (23 391 kg^−1^ dw [29]), Lake Michigan nearshore and offshore (65.2 kg^−1^ dw [28]) and Lake Superior beaches (65 kg^−1^ dw [30]). These values indicate that stormwater ponds are indeed critical transient sinks for MPs. The ponds are not considered permanent sinks because the sediment is removed when the basins are almost full. The ponds are dredged and the sediment is tested for chemical pollutants. If the pollutant levels are below maximum allowable concentrations, the sediment may be used as construction fill material where the MPs would become trapped in a potential permanent sink, or the sediment may be added to agricultural land, where the MPs could be washed into water bodies through runoff. If pollutant levels are high, the sediment is deposited in a landfill, which may be a permanent sink for MP debris unless the particles are small enough to percolate down through pore spaces and become part of the leachate that escapes landfill sites [31].

### (c) Main factors affecting microplastic abundances

The results indicate that the main factor controlling MP abundances is within-basin location, with the sediment forebays accumulating more MPs than the main basins. This result does not change whether all 15 ponds (*p*‐value = 0.0149) or only 13 (*p*‐value = 0.0058) are considered. Similarly, with the omission of anomalous sample 030-4, the pond inlets, which are located within the forebays, have a greater average abundance of MPs (20 ± 49 g^−1^ dw) compared to the main basin outlets (10 ± 17 g^−1^ dw). In their study of a constructed Australian floating wetland, Ziajahromi *et al*. [32] found that MPs were more abundant at the inlet rather than the outlet. Similarly, Öborn *et al*. [33] determined an average abundance of 23 600 kg^−1^ dw for inlet samples compared with 11 500 kg^−1^ dw for outlet samples from stormwater ponds in Sweden. Considering that the inlets are the main pathways through which stormwater runoff enters the ponds, we expected that MPs would be predominant in these locations. Although a portion of the low-density MPs may stay afloat and later settle in sediment of the main basin, the results suggest that the majority of the particles sink with the inflowing sediment or settle through the water column in the forebay.

We initially hypothesized that ponds that had not been dredged 10−15 years prior to sampling would contain more accumulated MPs than ponds that were dredged less than 10 years before sample collection. However, there was no relationship between MP abundance and last dredged date, as the two samples with the greatest MP concentrations, Oxford High Tech 030-4 and Hyde Park 3E 064-2, were in basins that were last dredged only 7 yr and 1 yr prior to sampling, respectively. In hindsight, this finding is not surprising because using a grab sampler removes the top 20 cm of sediment, but nothing that is buried deeper. An influence of the last dredged date would only be recorded if no new sediment had been deposited since the last dredging period.

Although there was no significant difference between MP abundance and pond orientation, N-S-trending ponds produced a greater average concentration than NE-SW, E-W and NW-SE ponds. This finding is not related to prevailing wind direction, as the wind mainly blows from west to east in London, Canada, for most of the year. In addition, one of the ponds trending N-S is Hyde Park 3E, which is actually composed of two ponds that connect over a narrow berm only when the water level is high. It is thus unlikely that long-axis orientation has any effect on the accumulation of MPs in stormwater ponds.

Local land use appears to have had a greater influence on MP abundances than pond orientation (figure 7B). When considering all 15 ponds, the average abundances of MPs were 6 ± 7 g^−1^ dw for residential ponds, 6 ± 11 g^−1^ dw for open ponds, 19 ± 22 g^−1^ dw for construction ponds and 870 ± 2662 g^−1^ dw for commercial/industrial ponds. Even with the omission of Oxford High Tech 030-4 (electronic supplementary material, fig. S1f), the commercial/industrial pond average was still the highest, with 28 ± 73 g^−1^ dw. Summerside and Hyde Park 3E were surrounded by housing construction and retail buildings, respectively, at the time of sampling, and their average MP abundances were the second and third highest of all 15 ponds studied.

### (d) Significance of microplastic types

The predominance of fragments in 14 ponds compared with other particle types agrees with results determined from other studies of stormwater pond sediment [24,25,34]. Separating black SBR particles from the general fragment category allowed us to determine that Oxford High Tech is the only pond with a sediment sample containing more SBR particles than fragments. SBR is mainly used in vehicle tyres, and therefore, the black rubber particles in the stormwater ponds are considered tyre wear particles (TWP). Ostini Goehler *et al*. [34] determined that 53% of MPs found in sediment from a São Paulo pond were composed of TWP, and Rasmussen *et al*. [26] showed that TWP were the most common MP type in three of the four ponds studied in Denmark. The industrial land use near Oxford High Tech pond could account for the excessive amount of TWP in sample 030-4. The total drainage area served by the pond is approximately 61 000 m^2^ and, in addition to one inlet discharging stormwater, the land surface within the drainage area slopes down 4 m to the pond over a distance of 200 m [35], which facilitates the transport of plastic debris by wind and stormwater runoff. Medical technology manufacturing and pharmaceutical manufacturing buildings are within 150 m of the pond (electronic supplementary material, fig. S1f). At one of these buildings, three loading docks are accessed by semi-tractor trailers with 18 wheels. In addition, a vehicle technology training building is located approximately 200 m from the pond. All of these activities could contribute SBR particles to Oxford High Tech stormwater pond.

We hypothesized that glitter would be most common near inlets, as these particles are composed of PET, which has a density of 1.38 g cm^−3^. The results instead show that glitter is found in sediment near inlets, outlets and open areas. This can be explained by the observed settling behaviours of natural sedimentary grains in water. Platy grains, like clay minerals, take longer to settle because they are most stable with their maximum surface area parallel to the water surface. The result is that platy grains have higher friction drag than more spherical particles of a similar size [36]. Like clay minerals, glitter particles are platy and therefore will settle once they are away from a region of turbulent flow. In most cases, the calm water in a stormwater pond is not associated with an inlet which, during a storm event, is usually the site of high hydraulic loading. Although hydraulic loading was not considered in the present study, it is probable that not all 15 ponds experience the same stormwater loads during high rain events, and this would account for the variations in within-pond distribution of glitter.

Comparing the average proportion of fibres in each pond with other particle types leads to six ponds with a greater than 20% fibre component: Stoney Creek 1N (32.6%), Duncairn (31.5%), Summerside (24.8%), South River (24.1%), Upland Hills (21.9%) and Talbot Village (21.8%) (figure 2). Five of the six ponds are considered residential, but Summerside is considered a construction-related pond. The latter, however, contains 36 houses within 100 m of the pond periphery, which is close to the average 44 houses for all residential ponds (table 1). Considering that plastic litter is directly related to the behaviours of humans, the higher relative fibre component in residential ponds compared to other land use ponds is not surprising. More houses generally equate to more people, and people wear clothing made of synthetic fibres, use fibrous building materials (e.g. insulation) and other textiles, such as carpets, draperies, tarpaulins and bedding.

Two types of fragments that were not separated from the general fragment category due to their inconspicuous nature were paint- and coating-related particles. These were, however, identifiable using FTIR, and the compositional results show that of the 357 fragments analysed, 25% were components of paints or coatings. These fragment types readily break down from surface degradation of numerous structures, such as houses, vehicles, sporting goods, tools and toys, and even dyed plastic packaging. Micro-fragments of paint and other coatings have been identified in countless studies (e.g. [16,29,37]) and, combined with the present study, support the contention that paints and coatings are prominent MP types in the environment. The numerous sources contributing to MP pollution in stormwater ponds are also evident in the 26 different polymer combinations that were identified using FTIR. This makes stormwater pond sediment potentially the best matrix in which to investigate sources and pathways of MPs into the environment.

## 5. Conclusions and future work

The results of this study show that sediment in urban stormwater ponds is a crucial sink for MP debris. MP abundances were greater in stormwater pond sediment compared to lake bottom, beach and river bottom sediment within the same watershed. The data show that the forebays of stormwater ponds are prevalent depositional sites for MPs compared to the main basins, with the mean abundances of all five morphologies (fragments, fibres, beads, glitter, black rubber) being greater in the former than the latter depositional site. The quantity and types of MPs identified in sediment of the London, Canada stormwater ponds are associated with local land use to some degree, wherein the extensive concentration of SBR fragments (TWP) in one sample can be related to proximal industrial activities, automotive technical training, parking areas and loading docks. Residential ponds contained the highest concentrations of fibres relative to other particle types, which can be related to the use of various textiles by a greater number of people in residential areas. Sediment samples collected from pond inlets had a greater average proportion of MPs compared with samples from the outlets and open pond areas, which suggests that many of the particles are sinking within the forebay rather than floating into the main basin.

Expansion of this study will include the addition of MP results from 13 more ponds, as well as macroplastic data from all 28 stormwater basins. This will enable relationships to be drawn between large and small plastic debris items and their common sources. In addition, further subdivision of the fragment category into films would enable determination of the proportion of fragments degraded from plastic bags and other film packaging. The final future results will guide the municipal government in deciding where to focus clean-up efforts and also inform policy regarding the management of different types of MP pollution sources.

## Data Availability

Data are included as supplementary files. Supplementary material is available online [38].
